# Supplementary figures and images for: Long-term outcomes after discontinuing biological drugs and tofacitinib in patients with rheumatoid arthritis: A prospective cohort study
Source: PLoS One. 2022 Jun 23;17(6):e0270391. doi: 10.1371/journal.pone.0270391 (PMC9223309; doi:10.1371/journal.pone.0270391)

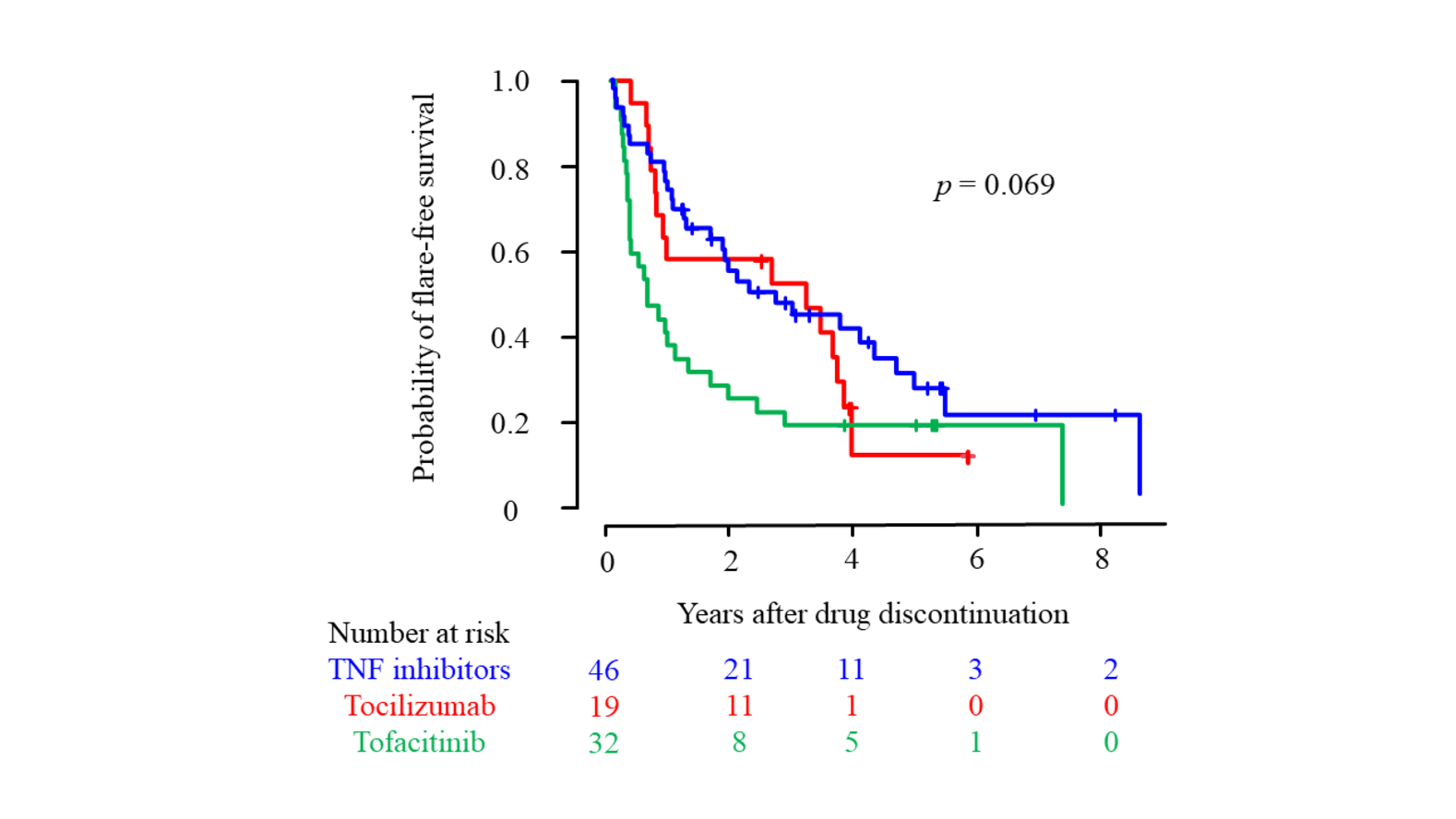

Supplement: S1 Fig — Kaplan–Meier plots for the proportion of patients who experienced no disease flare during follow-up are shown for patients who discontinued TNF inhibitors, tocilizumab, or tofacitinib. The flare-free probability among the patient groups was compared using the log-rank test with the post hoc Holm’s procedure. The p values were as follows: p = 0.069 for a comparison among the three groups; p = 0.030 for tofacitinib versus TNF inhibitors; p = 0.14 for tocilizumab versus tofacitinib; and p = 0.52 for TNF inhibitors versus tocilizumab. Median time to disease flare (95% CI) was 2.2 years (1.0–3.4 years) for TNF inhibitors, 3.1 years (0.0–6.3 years) for tocilizumab, and 0.6 years (0.2–1.0 years) for tofacitinib. Numbers below this figure represent the number of patients remaining in the analysis. bDMARDs, biological disease-modifying antirheumatic drugs; TNF, tumor necrosis factor; CI, confidence interval. (TIF) [file pone.0270391.s001.tif]
